# Two major chromosome evolution events with unrivaled conserved gene content in pomegranate

**DOI:** 10.3389/fpls.2023.1039211

**Published:** 2023-03-13

**Authors:** Zeynal Akparov, Sabina Hajiyeva, Mehraj Abbasov, Sukhjiwan Kaur, Aladdin Hamwieh, Alsamman M. Alsamman, Elchin Hajiyev, Sevda Babayeva, Vusala Izzatullayeva, Ziyafat Mustafayeva, Sabina Mehdiyeva, Orkhan Mustafayev, Ilham Shahmuradov, Peter Kosarev, Victor Solovyev, Asaf Salamov, Abdulqader Jighly

**Affiliations:** ^1^ Genetic Resources Institute, Ministry of Science and Education, Baku, Azerbaijan; ^2^ Research Institute of Fruit and Tea, Ministry of Agriculture, Guba, Azerbaijan; ^3^ Agriculture Victoria, Department of Jobs, Precincts and Regions, AgriBio, Centre for AgriBioscience, Bundoora, VIC, Australia; ^4^ Department of Biotechnology, International Centre for Agricultural Research in the Dry Areas(ICARDA), Giza, Egypt; ^5^ Department of Genome Mapping, Agriculture Research Center (ARC), Agricultural Genetic Engineering Research Institute (AGERI), Giza, Egypt; ^6^ Institute of Molecular Biology and Biotechnologies, Ministry of Science and Education, Baku, Azerbaijan; ^7^ Institue of Biophysics, Ministry of Science and Education, Baku, Azerbaijan; ^8^ Softberry Inc., Mount Kisco, NY, United States; ^9^ U.S. Department of Energy Joint Genome Institute, Lawrence Berkeley National Laboratory, Berkeley, CA, United States

**Keywords:** chromosome evolution, evolution, genomics, pan-genome, pomegranate

## Abstract

Pomegranate has a unique evolutionary history given that different cultivars have eight or nine bivalent chromosomes with possible crossability between the two classes. Therefore, it is important to study chromosome evolution in pomegranate to understand the dynamics of its population. Here, we *de novo* assembled the Azerbaijani cultivar “Azerbaijan guloyshasi” (AG2017; 2n = 16) and re-sequenced six cultivars to track the evolution of pomegranate and to compare it with previously published *de novo* assembled and re-sequenced cultivars. High synteny was observed between AG2017, Bhagawa (2n = 16), Tunisia (2n = 16), and Dabenzi (2n = 18), but these four cultivars diverged from the cultivar Taishanhong (2n = 18) with several rearrangements indicating the presence of two major chromosome evolution events. Major presence/absence variations were not observed as >99% of the five genomes aligned across the cultivars, while >99% of the pan-genic content was represented by Tunisia and Taishanhong only. We also revisited the divergence between soft- and hard-seeded cultivars with less structured population genomic data, compared to previous studies, to refine the selected genomic regions and detect global migration routes for pomegranate. We reported a unique admixture between soft- and hard-seeded cultivars that can be exploited to improve the diversity, quality, and adaptability of local pomegranate varieties around the world. Our study adds body knowledge to understanding the evolution of the pomegranate genome and its implications for the population structure of global pomegranate diversity, as well as planning breeding programs aiming to develop improved cultivars.

## 1 Introduction

Pomegranate (*Punica granatum* L.) is one of the oldest and most popular fruit plants for its nutritional, medicinal, and industrial values. The tree has a unique plasticity to different environmental conditions and soil profiles ranging from tropical to subtropical or even temperate zones (Chandra et al., 2010), with the ability to survive in extreme temperatures above 44°C and below −12°C (Westwood, 1978). Pomegranate is one of the first domesticated fruit crops that might have originated in the most distant prehistoric centuries, estimated at least 4 millennia (Levin, 2006; Still, 2006). Vavilov attributed the origin of the pomegranate to the Central Asian center of origin for cultivated plants. The presence of large populations of wild pomegranates in Azerbaijan, Turkey, Iran, Afghanistan, Turkmenistan, and India confirms that the core center of origin and cultivation/domestication of the pomegranate is the Middle Eastern mega-center (Levin, 2006; Holland et al., 2009). It was previously proposed that the domestication of pomegranates occurs independently in different regions, not only in the centers of origin (Narzary et al., 2021). At the population level, Luo et al. (2020) found high genetic differentiation between soft-seeded and hard-seeded cultivars using a set of 26 cultivars.

The evolution of *P. granatum* is unique as different cultivars have different numbers of diploid chromosomes. The first cytological study on pomegranates indicated the presence of eight bivalents (2n = 16) in metaphase 1 of meiosis (Morinaga et al., 1929). Multiple subsequent studies also concluded that the diploid chromosome number is 2n = 16 (Nath and Randhawa, 1959; Sheidai and Noormohammadi, 2005; Levin, 2006). However, other studies found that different wild and cultivated pomegranate trees had a diploid chromosome number of 2n = 18 (Kostoff et al., 1935). With decreasing sequencing costs, considerable attention has recently been paid to the genomes of previously understudied plants, including pomegranates. Currently, four reference genomes are available on NCBI, including the two traditional Chinese cultivars ‘Taishanhong’ (2n = 18), which is semi-soft seeded (Chater et al., 2020; Gasic et al., 2020), ‘Dabenzi’ (2n = 18), which is hard-seeded, and the soft-seeded Tunisian cultivar ‘Tunisia’ (2n = 16), which has been widely adapted in China (Luo et al., 2020; Chen et al., 2022). Another soft-seeded Indian cultivar, ‘Bhagawa’ (2n = 16), was recently published as a reference originated from the pomegranate center of origin (Roopa Sowjanya et al., 2022). However, another hard-seeded reference genome originated from the pomegranate center of origin is required to fully understand the evolutionary history of the pomegranate. Moreover, the availability of multiple genomes facilitates the development of graph references and pan-genomes in which core genes that exist in all individuals as well as dispensable genes that are represented in some individuals are defined. These graph references are important to accurately identify structural variations such as presence and absence variations as well as copy number variations.

In the present study, we carried out *de novo* sequencing and a transcriptome analysis of six organs of the “Azerbaijan guloyshasi” (AG2017) pomegranate variety, as well as re-sequencing of six pomegranate varieties at coverage >10×. We performed a pan-genome study on the obtained re-sequencing data using the “map-to-pan” strategy (Wang et al., 2018), as well as the five available reference genomes and found highly conserved genetic content across cultivars. We carried out a comprehensive comparative analysis with Bhagawa, Taishanhong, Tunisia, and Dabenzi to study the unique evolutionary history of pomegranate chromosomes, and we detected two major chromosome evolutionary events. We also revisited the results of Luo et al. (2020) on the divergence between soft- and hard-seeded pomegranates after increasing the population size with the re-sequenced individuals in the present study to increase the accuracy of detecting genomic regions that differentially affect both seed types.

## 2 Materials and methods

### 2.1 Plant material and nucleic acid extraction

Leaf tissues from multiple pomegranate genotypes (**Table S1**) obtained from the collection garden of the Genetic Resources Institute of the Azerbaijan National Academy of Sciences were used for DNA extraction. For RNA-Seq, multiple tissue sources, including leaves, stems (new and old), petals, flowers, and roots, were harvested at various time points. The harvested tissue was snap-frozen in liquid nitrogen and stored at −80°C until performing DNA/RNA extractions. All plant materials can be requested from the Genetic Resources Institute, Ministry of Science and Education, Azerbaijan.

DNA extraction was performed using the DNeasy^®^ Plant Mini Kit (Qiagen, Hilden, Germany) as per the manufacturer’s instructions. Quality control was performed by resolving the DNA sample on a 0.8% (w/v) agarose gel. For RNA extraction, the method described by Zarei et al. (2012) was followed except for resuspending the RNA pellet in 20 µl of RNase-free TE buffer. The extracted RNA samples were resolved on a 1.2% (w/v) denaturing agarose gel to assess the integrity of the nucleic acid. Both the isolated DNA and the total RNA samples were quantified using a spectrophotometer (Thermo-Scientific, Wilmington, DE, USA) at wavelength ratios of A260/280 and A260/230.

### 2.2 Defining the number of chromosomes

Cytogenetic studies were performed on four *P. granatum* local and two introduced cultivars (Gizili (Azerbaijan), Purpursid (USA), Goynar (Azerbaijan), Valas (Azerbaijan), Achygdona (Uzbekistan), and Fatima (Azerbaijan)) in 2021. Forty to 80 young flower buds (long- (bisexual), middle- (intermediate), and short (male) pistiled) containing meiotic divisions in the PMCs (pollen mother cells) were randomly collected between 9 and 12 a.m. and fixed in glacial acetic acid: ethanol 96% (1:3) for 24–72 h. After thorough washing, the experimental materials were transferred to 70% ethanol and placed in a cool place (+5°C) to be used for subsequent studies. Anthers were stained with 2% aceto-carmine, dissected out, and squashed on microscopic slides in 45% acetic acid medium (Darlington and Lacour, 1969). The cover slip was placed on it and heated on a hot plate repeatedly to avoid boiling the cell suspension. After heating, the slides were pressed firmly to get a good spread of chromosomes. The slides were then sealed and observed under a microscope (Nikon Eclipse) with oil immersion lens.

### 2.3 Genome library preparation, sequencing, and assembly

For the genotype Azerbaijan guloyshasi, two paired-end libraries with 350 and 550 bp inserts were prepared using the Illumina TruSeq^®^ DNA PCR-Free Library Prep kit as per the manufacturer’s instructions. To further generate a higher diversity of fragments with 2–15 kb inserts, one mate pair library was prepared using Illumina’s Nextera^®^ Mate Pair Library Prep kit following the Gel-Free protocol as detailed by the manufacturer. The libraries were assessed on the Agilent TapeStation 2200 platform with High Sensitivity D1000 ScreenTape (Agilent Technologies, Santa Clara, CA, USA) following the manufacturer’s protocol. The assessed libraries were further quantified using the KAPA library quantification kit (KAPA Biosystems, Boston, USA) according to the protocol described by the manufacturer prior to subjecting them to sequencing on three lanes of the HiSeq 1500 system (Illumina Inc., San Diego, USA). To minimize sequencing errors for assembly, raw reads from the Azerbaijan guloyshasi genotype were filtered for adaptor contamination and poor quality. Any reads containing more than three consecutive Ns or more than three nucleotides with a PHRED score of ≤20 or a median PHRED score of <20 or a read length of <50 nucleotides for paired end and 25 nucleotides for mate pair data were trimmed. *De novo* assembly was performed using SOAPdenovo software 1.05_127mer (Li et al., 2008). Reads were first assembled from the short insert size (c. 300–500 bp) libraries into contigs using de Bruijn graph k-mer overlap information. The long insert sizes of mate-paired libraries (≥2 kb) were not initially used, as the chimeric reads common to such libraries can generate incorrect sequence overlaps. To avoid this problem, a hierarchical assembly method was used through step-by-step scaffold construction by data addition from each library separately ranked from smallest to largest according to insert size. The number of reads can be found in **Table S1**.

### 2.4 RNA-Seq library preparation, sequencing, and assembly

A total of six RNA-Seq libraries were prepared with the Illumina TruSeq^®^ Stranded RNA LT Kit according to the protocol described by the manufacturer. The libraries were assessed on the Agilent TapeStation 2200 platform with D1000 ScreenTape (Agilent Technologies, Santa Clara, CA, USA) following the manufacturer’s protocol. Each RNA-Seq library was prepared with a unique indexing primer, and all six libraries were multiplexed at an equimolar concentration obtained from the TapeStation to generate a single pool. The multiplexed pooled sample was quantified using the KAPA library quantification kit (KAPA Biosystems, Boston, USA) according to the protocol detailed by the manufacturer. The quantified sample was subjected to pair-end sequencing using the HiSeq 3000 system (Illumina Inc., San Diego, USA).

The raw sequence reads were filtered using a custom perl script and Cutadapt v1.9 (Marcel, 2011). Low-quality reads (reads with >10% bases with Q ≤20) and adaptor sequences were removed from the sequenced reads. Data trimming involved the removal of reads that had three or more consecutive unassigned Ns with a PHRED score of ≤20. Prior to the *de novo* transcriptome assembly step, sequence reads that were less than 50 bp were also discarded. The filtered data was primarily assembled using the transcriptome assembler, SOAPdenovo-TRANS (Xie et al., 2014), with a k-mer size of 101. Further, bubble, fork, and complex loci obtained from SOAPdenovo-TRANS assembly were further combined using the CAP3 assembler (Huang and Madan, 1999) with a minimum overlap of 50 bp and 95% identity to generate longer sequences. Transcripts having a total length of less than 240 bp were removed, as these were considered shorter than the length of a single pair of the sequence. The assembled transcripts were aligned with BLASTX against the NCBI non-redundant (Nr) protein database and the UniRef100 database version 1.0 with an E-value of <10^−10^.

### 2.5 Pairwise whole genome alignment

Pairwise whole genome alignment between different *de novo* assembled reference genomes was conducted using MUMmer V3.23 (Delcher et al., 2002) with a maximum gap length of 500 bp and a minimum cluster length of 100 bp. The resulted coordinates were visualized using the R package “circlize” (Gu et al., 2014) to present the synteny between chromosomes/scaffolds for different reference genomes.

### 2.6 Multiple whole genome alignment and phylogeny

Whole genome sequence alignment for the five *de novo* assembled cultivars Azerbaijan guloyshasi, Bhagawa, Taishanhong, Tunisia, and Dabenzi was conducted using the software MUGSY V1.2.3 (Angiuoli and Salzberg, 2011) with its default parameters. Scaffolds with a length of <10 kb were excluded from this analysis. Only aligned segments that existed in all five genomes were used to avoid bias due to incompleteness of genomes (e.g., the assembly size for Taishanhong was only 274 Mb while the estimated genome size was 322.7 Mb). The phylogenetic tree was constructed using IQ-TREE software V1.6.5 (Nguyen et al., 2015) with the model finder option “mtree” which finds the best model from 286 DNA models. The best model was “GTR + F + I” which represents the General time reversible model (GTR; Tavaré, 1986) with unequal rates (+I: estimate the proportion of invariable sites to consider the heterogeneity rate between lineages and across sequence positions) and unequal base frequencies (+F: Empirical state frequency observed from the data). Phylogenetic trees were visualized with FigTree v1.4.4 software (https://github.com/rambaut/figtree/).

### 2.7 Pan-genome assembly and annotation and graph reference construction

Six cultivars were selected for pan-genome analysis and re-sequenced with coverage ranging between 10× and 80×. The dataset included four cultivars from Azerbaijan, one from Uzbekistan, and one from the USA, plus the *de novo* assembled genomes (Azerbaijan guloyshasi, Taishanhong, and Dabenzi). The reads for each cultivar were mapped to the Tunisia genome using the software BOWTIE2 V2.3 (Langmead and Salzberg, 2012). Unmapped reads were pooled and *de novo* assembled for each cultivar independently using MaSuRCA V4.0.2 software (Zimin et al., 2013) to produce new sequences that are not located in the Tunisia genome, and only contigs with size >1,000 bp were kept. Assembled contigs were compared to all available bacterial, plastid, and mitochondrial genomes on NCBI (downloaded on 21 August 2021). Only hits with E-values <10^−8^ and length >100 bp were kept. Contigs that had aggregated matches >25% of their lengths with >70% identity were filtered out and considered contamination. Remaining contigs were annotated using MAKER2 software (Holt and Yandell, 2011), while Augustus (Stanke et al., 2006) and SNAP (Schmid et al., 2003) software were used for *de novo* gene prediction using publicly available RNA and protein data for pomegranate (downloaded on 23, 8, 2021) as well as the RNAseq data produced in the present study. The cleaned and non-reference contigs were pooled, and redundant sequences were eliminated using the software CD-HIT (Li and Godzik, 2006). The remaining contigs were re-aligned to the Tunisia reference to ensure that they do not exist in the reference. These contigs and their annotations were pooled and added to the reference Tunisia to define the gene presence and absence variation among the cultivars and to build the graph reference. The graph reference was generated using vg V1.37 software (Garrison et al., 2018).

### 2.8 Gene presence and absence variation

Whole genome data for the eleven pomegranate cultivars (six re-sequenced cultivars as well as five *de novo* assembled genomes) were mapped to the Tunisia reference genome after adding the pooled non-reference contigs produced in the previous step using BBMap V35.85 software (Bushnell, 2014). The resulted BAM files were analyzed with the software SGSGeneLoss V0.1 (Golicz et al., 2015) to determine the gene presence and absence variation of the pooled genes.

### 2.9 Variant calling

Genotype likelihoods at each genomic position from the BAM files generated when aligning the reads of each cultivar to the Tunisia reference were calculated using the “mpileup” command implemented in bcftools (Li, 2011). The output was processed with the bcftools command “call” to generate a VCF file for each genome with the -m option (alternative model for multiallelic and rare-variant calling). Finally, the VCF files for all cultivars were merged with the “merge” command implemented in bcftools.

### 2.10 Population genetics analyses

The re-sequenced cultivars in the present study were added to the 26 cultivars reported in Luo et al. (2020). Linkage disequilibrium decays for soft-seeded and hard-seeded subpopulations were estimated as the r2 value (Hill and Robertson, 1968) calculated between each pair of loci located on the same chromosome with the R package “snpstats” (Clayton, 2015). The r2 values were plotted against the physical distance between each pair of loci, and the second-degree Loess smoothing line was fitted and plotted using R. Principal component analysis (PCA) was calculated with PLINK V1.9 software (Purcell et al., 2007), and the first two PCs were plotted with R. Population structure was inferred using the software ADMIXTURE (Alexander et al., 2009). A hundred replicates of the analysis were conducted, with the number of underlying subpopulations (K) ranging from 2 to 10 with 20 cross-validations. The optimal K value was defined as the lowest average cross-validation value over the 100 replicates, which was K = 3 (**Figure S2**). However, different replicates showed the lowest cross-validation value at K = 4, so we plotted the results for K = 2, 3, and 4. The phylogenetic tree was constructed using IQ-TREE software V1.6.5 (Nguyen et al., 2015) with the model GTR + ASC, which is designed to handle ascertainment bias in SNP data. The analysis was run with 1,000 bootstraps and 1,000 replicates of the SH-like approximate likelihood ratio test (Guindon et al., 2010).

Genomic loci that were differentiated between hard-seeded and soft-seeded subpopulations were selected to have the reference allele fixed (or have a maximum of one heterozygote cultivar) in one subpopulation while the other subpopulation had the alternative allele fixed (or have a maximum of one heterozygote cultivar). The advantage of repeating this analysis in our study over that reported in Luo et al. (2020) is that we included the soft-seeded cultivar “Purpursid,” which was genetically clustered with the hard-seeded cultivars. Therefore, it is expected to have fewer and shorter differentiated genomic regions between soft- and hard-seeded subpopulations compared to the results presented in Luo et al. (2020).

## 3 Results

### 3.1 Genome and RNA-Seq assembly for the cultivar Azerbaijan guloyshasi (AG2017)

Cytological studies on the studied pomegranate cultivars showed that they all possessed 2n = 16 chromosomes, and at diakinesis and M1, the association of 8II was noted (**Figure S1**). **Table S1** shows a summary of sequencing reads for each cultivar. Based on empirical testing for genome assembly, an optimal k-mer size of 91 was used for SOAPdenovo assembly. Initially, reads from short insert sizes were assembled into 908,448 contigs, with an N50 of 1,568 bp. After adding the long insert size mate-paired libraries, the final assembly consisted of 12,363 transcripts (>500 bp), representing a cumulative length of 371.6 Mbp with an N50 of 220,197 bp (**Table S2**). For RNA-Seq data, the optimal k-mer size of 101 was identified after empirical testing. Initial assembly resulted in 78,359 transcripts, representing a cumulative length of 67.7 Mbp with an N50 of 1,657 bp (**Table S2**). Following that, the CAP3 program was used to analyze and assemble a set of scaffolds that were identified as specific loci and contained multiple sequence entries described as forks, bubbles, or complexes. The final assembly consisted of 58,064 transcripts after filtering transcripts <241 bp, with a total assembly length of 56,497,814 bp and an N50 length of 1,561 bp (**Table S2**). All transcripts from this assembly were BLASTX against the non-redundant (Nr) and UniRef100 databases, allowing the identification of a total of 29,216 transcripts (50%) with significant matches to protein databases (28,972 and 29,019, respectively).

### 3.2 Synteny and phylogeny of *de novo* assembled genomes

**Figure 1:**
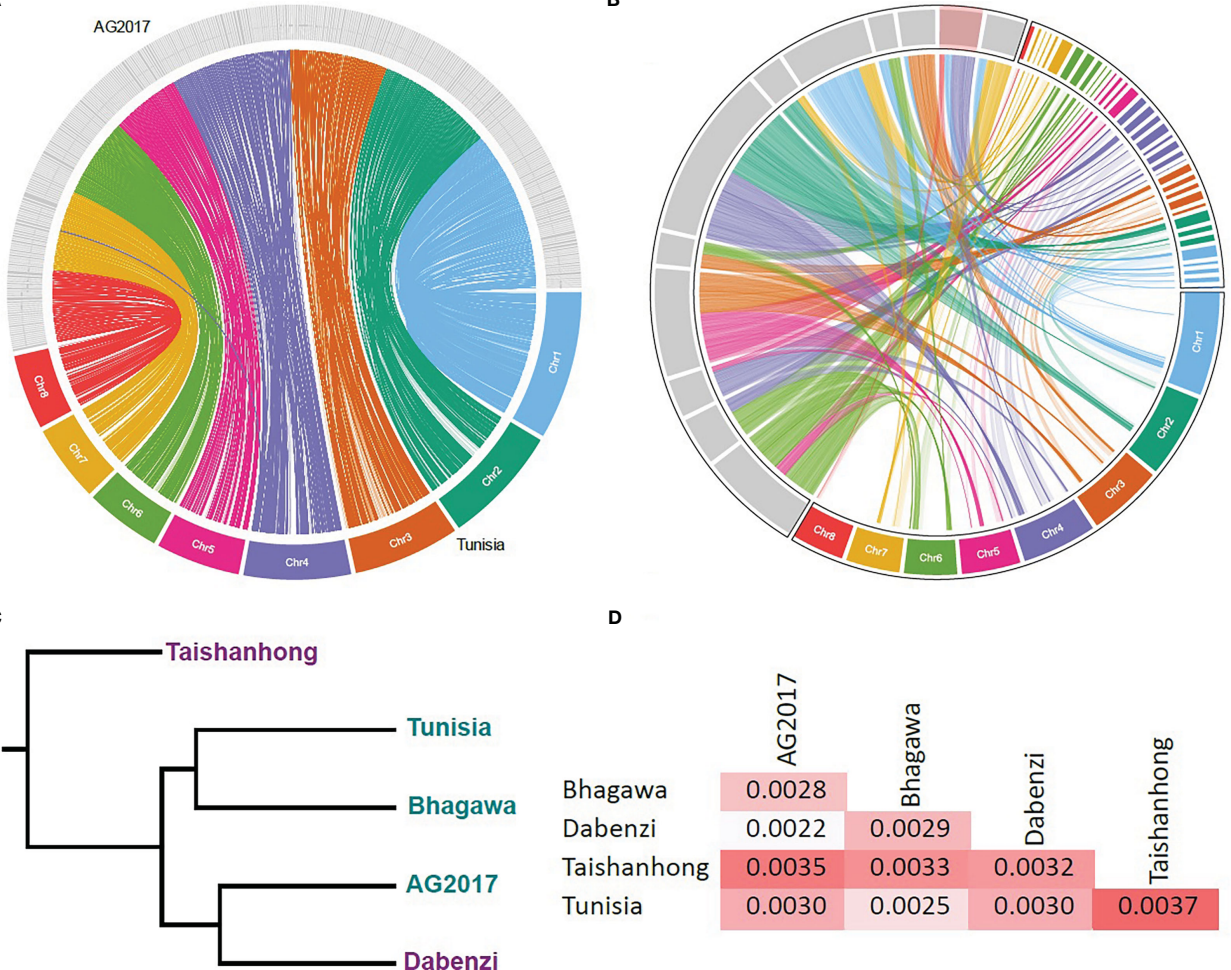
Comparative genomics of different *de novo* assembled genomes. **(A)** A circus plot showing the synteny between the Tunisia genome and the largest 400 scaffolds of the AG2017 genome; **(B)** the synteny between the Dabanzi and Tunisia genomes with 12 scaffolds of the Taishanhong genome that were aligned to different Tunisia chromosomes; highlighted red scaffold represents the scaffold MTJX01000087.1 which was aligned to three chromosomes; **(C)** whole-genome alignment-based phylogeny of the five genomes; purple IDs represent the genomes with nine diploid chromosomes while blue IDs represent the genomes with eight diploid chromosomes; **(D)** maximum likelihood distance matrix between the five genomes.

The genome of the AG2017 cultivar showed a very high synteny with the cultivar Tunisia, which was assembled to a chromosome level. All scaffolds were completely and continuously mapped to a single chromosome except for scaffold4026, which was split into the centromeric region of chromosome 4 and the telomeric region of chromosome 7. **Figure 1A** shows the relation between scaffolds >250 kbp (400 scaffolds) of the cultivar AG2017 and Tunisia chromosomes. These large scaffolds seem to be in the telomeric regions, while short scaffolds are concentrated more in the centromeric regions. These two cultivars have eight diploid chromosomes. Interestingly, they both showed very high synteny with the Chinese cultivar Dabanzi, which has nine pairs of chromosomes, as well as the Indian cultivar Bhagawa, which has eight pairs of chromosomes. However, when compared to the other Chinese reference with nine diploid chromosomes, “Taishanhong,” 11 scaffolds were mapped to two different chromosomes, while one scaffold, “MTJX01000087.1,” was mapped to three chromosomes (1, 4, and 8). **Figure 1B** presents the synteny between these scaffolds with the Dabanzi and Tunisia sequences. It is clear from the figure that the breakpoints of these scaffolds have been mapped to different locations on both genomes, and the synteny of these locations is matched on the Dabanzi and Tunisia genomes.

A whole genome alignment of the five cultivars (only scaffolds >10 Kbp) was conducted, and only genomic regions that were found in the five genomes were used to infer the phylogenetic tree. The total length of the matched sequences was 267.7 Mbp, which is equal to 98.9% of the total size of Taishanhong scaffolds with length >10 Kbp (270.8 Mbp) that were used in the analysis. Dabenzi and AG2017 have the highest relatedness among all cultivars (**Figures 1C, D**), followed by Tunisia and Bhagawa. This is a very interesting finding, giving that both cultivars have different numbers of chromosomes. Tunisia had an equal distance from both cultivars, while Taishanhong showed the highest distance from all cultivars. However, Taishanhong was most related to the Dabenzi cultivar, and they both have nine diploid chromosomes (**Figure 1D**).

### 3.3 Pan-genome of pomegranate

**Figure 2:**
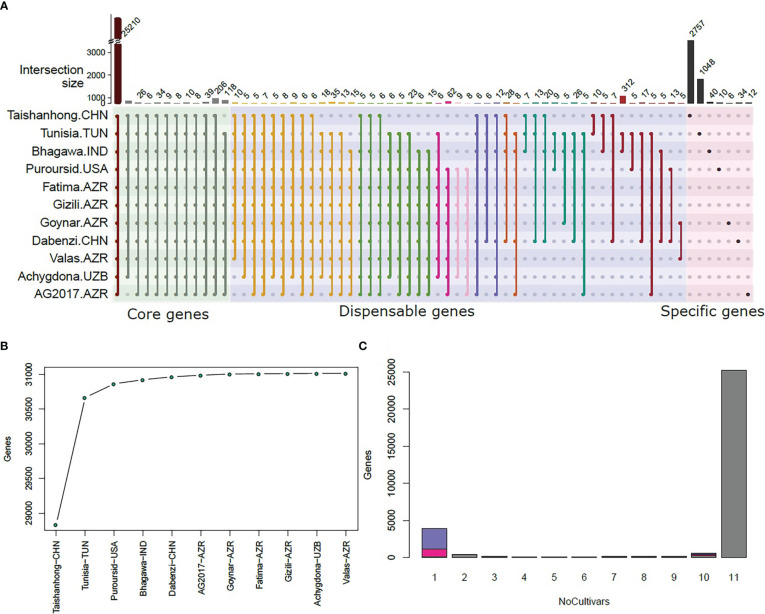
The pan-genome of pomegranate. **(A)** Histograms showing the core gene clusters (present in at least nine genomes), dispensable gene clusters (present in two to eight genomes), and lineage-specific genes (present in a single cultivar). Only combinations that have five genes or more were plotted; **(B)** the increase of the number of genes with increasing the number of cultivars; **(C)** the total number of genes versus the number of cultivars in which they exist. For categories 1 and 9, purple and pink colors represent the number of genes that existed or did not exist in Taishanhong and Tunisia cultivars, respectively.

The pan-genome dataset (six re-sequenced and four *de novo* assembled cultivars) added up to a total of 1,941 contigs to the Tunisia reference genome with an aggregated sequence length of 4.1 Mbp (**Table 1**). The number of contigs ranged from 25 for Achygdona to 1,001 for Taishanhong, with total lengths ranging between 34.6 Kbp and 1.8 Mbp, respectively (**Table 1**). These results indicated very limited insertion/deletion variation among the studied cultivars despite the wide geographical and botanical variations in the population. The variant summary between the re-sequenced panel and the Tunisia genome can be found in **Table S3**. The total number of genes in the pan reference was 31,016 of which 25,210 (81.3%) existed in all eleven cultivars. A total of 552 (1.8%) were missing from only one cultivar, of which 324 (58.7%) were missing from Taishanhong and Tunisia (**Figure 2C**). The previous two categories were considered core genes (83.1%) that existed in almost the whole population (**Figure 2A**). On the other hand, 3,916 (12.6%) genes were cultivar-specific genes that existed in a single cultivar only, of which the majority (3,805, 97.2%) belong to the cultivars Taishanhong and Tunisia (**Figure 2C**). These genes were assumed to be cultivar-specific genes. The remaining 1,338 genes (4.3%) existed in different numbers of cultivars, ranging between two and nine cultivars, which were considered the dispensable set of genes. Interestingly, the gene content of the cultivars Taishanhong and Tunisia represents 98.9% (30,667) of the whole pan-genome genes, while the cultivar Purpursid added another 198 (0.64%) new genes to the set. The remaining eight cultivars combined added only 151 (0.49%) genes to the gene content of the reference pan-genome (**Figure 2B**).

**Table 1:** Sequences existed in each cultivar that do not exist in the Tunisia reference genome.

Cultivar	Contigs	Size (bp)	Avr (bp)
**Achygdona**	25	34,582	1,383
**AG2017**	441	796,992	1,807
**Bhagawa**	269	773,132	2,874
**Dabenzi**	201	602,273	2,996
**Fatima**	192	355,141	1,850
**Gizili**	107	253,856	2,372
**Goynar**	114	220,171	1,931
**Purpursid**	363	753,291	2,075
**Taishanhong**	1,001	1,797,254	1,795
**Valas**	51	78,397	1,537
**Total**	2,114	4,104,151	1,941

### 3.4 Soft- and hard-seeded subpopulations

**Figure 3:**
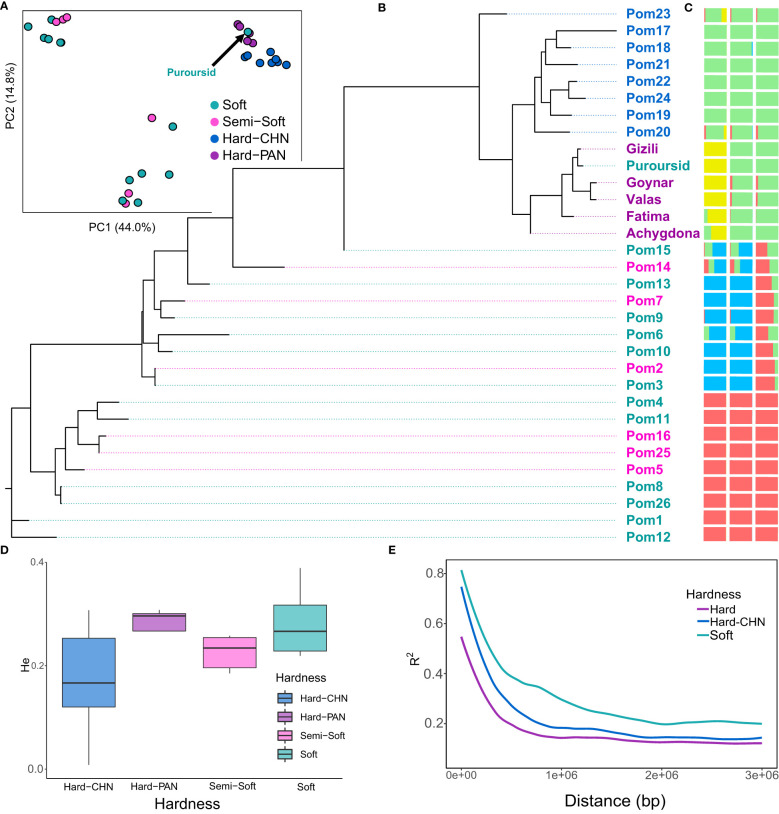
The population genetics of the re-sequenced cultivars. **(A)** the principal component analysis for the whole populations; **(B)** the phylogenetic tree for all cultivars; **(C)** the ADMIXTURE analysis at K = 2 (right), K = 3 (middle), and K = 4 (left); **(D)** the heterozygosity variation in different subpopulations; **(E)** linkage disequilibrium decay for different subpopulations.

Our re-sequenced panel was added to the 26 cultivars that were previously used by Luo et al. (2020), which included eight hard-seeded cultivars originated from China, to have a deeper understanding of the domestication of pomegranates and the diversion between soft- and hard-seeded subpopulations. Like Luo et al. (2020), hard-seeded cultivars seem to diverge from semi-soft and soft-seeded cultivars. However, the soft-seeded cultivar “Purpursid” from our re-sequenced panel was clustered with the re-sequenced hard-seeded panel (PAN) in the PCA, phylogeny, and ADMIXTURE analyses (**Figures 3A–C**). The PAN panel in the present study (all hard-seeded except Purpursid) formed an independent cluster that is most related to the hard-seeded cultivars of Luo et al. (2020). The hard-seeded cultivars from the PAN panel showed the highest heterozygosity over all other groups (**Figure 3D**) and adding them to the hard-seeded subpopulation had a substantial influence on reducing LD decay, especially within a distance of <1 Mbp (**Figure 3E**). The Azerbaijani cultivar Fatima and the Uzbekistani cultivar Achygdona showed some admixture with the Chinese hard-seeded cultivars, while the Chinese cultivar Pom23 showed some admixture with the re-sequenced group. Two soft and semi-soft Italian cultivars (Pom14 and Pom15) and one soft cultivar from the USA (Pom6) that belong to one of the soft-seeded subpopulations (colored blue in **Figure 3C**) showed some admixture with the Chinese hard-seeded cultivars.

Having a soft-seeded cultivar with a genetic background related to the hard-seeded cultivars in the present dataset, “Purpursid,” increased the power of detecting genes that control seed hardness and reduced the confounding effect of population structure. Our analysis detected 10 genomic regions that are highly differentiated between the soft- and hard-seeded subpopulations, of which four were common with the 24 regions detected in Luo et al. (2020). The description of these genomic regions and the annotations of the closest genes to them are presented in **Table 2**. Of these, chromosome 5 seems to have the largest region between 11.36 Mbp and 11.50 Mbp, which involves a cluster of four candidate genes. All SNPs within this region had *R^2^* values >0.95. Many of the detected genes along the genome have dehydrogenase and CoA synthase functions, as well as other seed development and cell wall-related activities.

**Table 2:** Genomic regions that showed high diversion between soft- and hard-seeded cultivars. Loci with underlined chromosome positions are the loci that are common with the one detected in Luo et al. (2020).

Chr	Position	Gene	Start	End	Annotation
Chr1	11,265,285	LOC116195387	11,267,388	11,268,657	late embryogenesis abundant protein Lea5-like
Chr2	**34,332,768**	LOC116196913	34,333,038	34,335,995	NRT1/PTR FAMILY 2.10-like
Chr3	38,368,453	LOC116201947	38,380,754	38,382,844	3-ketoacyl-CoA synthase 12-like
Chr4	16,546,459	LOC116206080	16,562,644	16,563,970	secoisolariciresinol dehydrogenase-like
Chr5	**11,360,872 to 11,495,723**	LOC116207452	11,374,356	11,377,493	epoxide hydrolase A-like
		LOC116207451	11,383,152	11,386,479	lipoamide acyltransferase component of branched-chain alpha-keto acid dehydrogenase complex
		LOC116208343	11,442,930	11,449,322	peroxisomal fatty acid beta-oxidation multifunctional protein MFP2
		LOC116208154	11,485,969	11,487,326	probable 3-ketoacyl-CoA synthase 21
Chr6	5,174,766	LOC116210304	5,168,216	5,171,790	endoglucanase 2-like
Chr6	**23,438,431**	LOC116212014	23,425,207	23,428,729	F-box/kelch-repeat protein At1g67480
Chr7	**7,224,200**	LOC116214184	7,221,650	7,223,513	F-box protein PP2-B15-like
Chr7	20,387,668	LOC116213092	20,395,581	20,397,305	probable pectinesterase 55
Chr7	20,939,223	LOC116213519	20,936,244	20,940,033	pathogenesis-related thaumatin-like protein 3.5

## 4 Discussion

The basic number of chromosomes in the genus Punica is 2n = 14, with seven bivalent chromosomes. However, *P. granatum* evolved with 2n = 16 or 2n = 18 chromosomes with eight or nine bivalent chromosomes (Shilkina, 1973; Sheidai and Noormohammadi, 2005; Levin, 2006). We *de novo* assembled the genome of the cultivar “Azerbaijan guloyshasi” (AG2017), which has 2n = 16 chromosomes. Interestingly, we found that the closest cultivar to it is the Chinese cultivar Dabenzi (2n = 18), despite the presence of other cultivars “Tunisia” and “Bhagawa” with (2n = 16) chromosomes, which may indicate high gene flow between their populations. It was previously thought that pomegranates were first introduced to China from central Asia ~200 BC along the Silk Road (Yuan et al., 2007; Chandra et al., 2010). Tunisia was the closest to Bhagawa, meaning that it could have been geographically transferred (to Tunisia) from the Bhagawa population. Tunisia was introduced to China in 1986 (Chen et al., 2022), which might be a short period to progress sufficient gene flow to reduce its divergence from other populations, unlike Dabenzi, which is known to have a cultivation history of over 300 years in China (Chater et al., 2020). Taishanhong (2n = 18) showed the highest distance with the other cultivar with 2n = 18 chromosomes, Dabenzi, which means that it was evolved from a cultivar related to it with 2n = 18 chromosomes. The mother tree of Taishanhong was first found on Mount Tai in China in 1984, and it was over 140 years old (Ran et al., 2015). Despite the high level of chromosomal rearrangements in Taishanhong, it still has high synteny with the other genomes, given that >98.9% of its genome was aligned to the other four *de novo* assembled genomes.

Gene flow between pomegranate cultivars with different chromosome numbers is possible. For example, Jalikop (2007) made crosses between the cultivar “Double Flower” and the cultivar “Ganesh” without discussing the number of chromosomes in both cultivars. Nath and Randhawa (1959) earlier reported that the cultivar Double Flower had 2n = 18 chromosomes, while the cultivar Ganesh had 2n = 16 chromosomes. Such crosses imply high synteny between the eight and nine diploid chromosome sets where they are fertile. Our results indicated that Dabenzi (2n = 18) has a very high level of synteny with AG2017, Bhagawa, and Tunisia (2n = 16). Dabenzi was assembled to a pseudochromosome scale (Qin et al., 2017), but unfortunately, the scaffold order was not published and is not available to the public. However, the size of the largest Tunisia chromosome (chromosome 1) is larger than 55 Mbp, while the size of the pseudochromosomes of the Dabenzi cultivar ranges between ~10 Kbp and ~40 Kbp. This could imply that the variation in chromosome number for some cultivars is just a result of splitting a single chromosome into two chromosomes. However, in the case of Taishanhong, it seems that its extensive chromosome rearrangements affected its fertility when crossed with other cultivars. This is because Taishanhong has the highest distance from other cultivars, even though it seems to be the most recently diverged cultivar in our dataset, as discussed earlier. This restricted crossability limits gene flow to other cultivars, leading to such a high distance. Nevertheless, it is important to ensure in future research that these rearrangements in the Taishanhong genome are true rearrangements and not assembly errors. It is also important to confirm if Taishanhong can be crossed with other cultivars or not.

The availability of multiple sequenced genomes as well as re-sequenced cultivars facilitates studying the pan-genome of pomegranate. Our results showed that the two cultivars Tunisia and Taishanhong almost represented the whole pan-genome gene content (~98.9%) of the 11 cultivars, despite multiple chromosomal evolutionary events. The cultivar Taishanhong could have more novel gene contents given that its estimated genome size was 336 Mbp while the assembled scaffold size was 274 Mbp (~81.5%) (Yuan et al., 2018). Therefore, it will be important to complete its genome and to further search for new diverse pomegranate genetic resources. To the best of our knowledge, such highly conserved gene content was only reported on cultivated soybean (Torkamaneh et al., 2021), but their results were contradictory with earlier reports on soybean that included exotic and landrace soybean germplasms (Li et al., 2014; Liu et al., 2020). In the case of pomegranates, wild trees are assumed to be as diverse as cultivated ones (Evreinoff, 1957) or even less diverse (Narzary et al., 2021). This is because many cultivars were selected from wild trees concurrently over the whole wild range, and gene flow is possible between both germplasms (Holland et al., 2009). Nevertheless, extending the present study to include wild and cultivated pomegranates from worldwide collections in the future is required. This will confirm if the limited sequences that exist in cultivars other than Tunisia (**Table 1**) are limited to the studied cultivars in the present study or if that is a common feature in global pomegranate germplasm.

The previous study by Luo et al. (2020) reported a very high divergence between soft- and hard-seeded cultivars. Given that the hard-seeded cultivars have faster LD decay compared to the soft-seeded ones, it is more likely that they are the wild type, and they have a larger effective population size. Luo et al. used a small hard-seeded set of eight Chinese cultivars that have limited diversity (compared to the whole hard-seeded population), which was inferred from their low heterozygosity. The PAN set seems to have the highest heterozygosity, and it further contributed to accelerating the LD decay of the whole hard-seeded population (**Figures 3D, E**), which means that it reserves higher genetic diversity. The Purpursid cultivar has soft seeds, but it was clustered with the PAN set. Therefore, the diversion between soft- and hard-seeded cultivars seems to be aided by breeding activities rather than occurring naturally.

Spreading pomegranates around the world also seems to happen through multiple routes. The cultivar Pom23, which originated in Xinjiang, China, showed a level of admixture with the PAN set, which might be a result of being geographically closer to Uzbekistan and Azerbaijan than the other Chinese cultivars. Moreover, the two Italian cultivars Pom14 and Pom15 were clustered with another seven cultivars from the USA (blue color in **Figure 3C**). They both showed some admixture with the population of the Chinese hard-seeded cultivars, which means that they were migrated to Italy from China, and then another migration happened from Italy to the USA.

The inclusion of the cultivar Purpursid in our dataset has the power to shorten the differentiated genomic regions between soft- and hard-seeded subpopulations compared to the results presented in Luo et al. (2020), leading to more precise detection of genes controlling seed hardness. Luo et al. (2020) identified two main genes (PgL0145810 and PgL0145770) that affect sucrose allocation and transport as candidate genes controlling seed hardness. However, comparing the sequence of these genes and their flanking regions (10 Kbp upstream and downstream of the genes) between Purpursid and the hard-seeded *de novo* assembled genomes did not show any unique variation in Purpursid. Therefore, further investigations of these genes should be considered to examine if the causal variant is an epigenetic variant or if seed softness in Purpursid is controlled by a different set of genes.

Genes affecting biosynthesis, or the degradation of cell walls, are key factors affecting seed hardness (Cosgrove, 2005; Tateishi et al., 2005; Niu et al., 2018). In our study, two genes were annotated as 3-ketoacyl-CoA synthase, which is essential in the biosynthesis of very long-chain fatty acids (Niu et al., 2018). On the other hand, another gene was annotated as peroxisomal fatty acid β-oxidation, which involves cytosolic acetoacetyl-CoA thiolase that catalyzes acetoacetyl-CoA synthesis (Lange et al., 2004). Previous studies showed that most 3-ketoacyl-CoA synthase genes can significantly increase the thickness of cell walls (Xiao et al., 2016), which affects seed hardness. Another gene encodes secoisolariciresinol dehydrogenase-like enzymes that are involved in lignan biosynthesis (Gabr et al., 2021). Enzymes like pectinesterase can soften fruit tissues by decomposing pectic polysaccharides in the cell wall of the seed (Inari et al., 2002). The endoglucanase enzyme can loosen the xyloglucan-cellulose network and hydrolyze cellulose within the cell wall (Jara et al., 2019). Zhang et al. (2010) suggested that a higher level of epoxide hydrolase in the fruit could increase the level of lactones, which increases seed hardness (Qin et al., 2020). Deng et al. (2018) reported an F-Box protein that was associated with fruit firmness.

## Data availability statement

The datasets presented in this study can be found in online repositories. The names of the repository/repositories and accession number(s) can be found below: https://www.ncbi.nlm.nih.gov/genbank/, GCA_002837095.1.

## Author contributions

ZA, MA, and AH conceived the study. AJ, MA, AH, and SK planned and structured the paper. SH, ZM, SM, and VI prepared the plant materials and performed cytogenetic analysis. EH, SB, and SH extracted DNA/RNA and done library construction. SK and MA performed sequencing. AJ, AA, SK, IS, OM, AS, PK, and VS carried out assembly and bioinformatics/statistical analyses and drew the figures. AJ, MA, SK, AA, and AH wrote the manuscript. All authors listed have made a substantial, direct, and intellectual contribution to the work and approved it for publication.
