# Supplementary material for: Two major chromosome evolution events with unrivaled conserved gene content in pomegranate
Source: Front Plant Sci. 2023 Mar 13;14:1039211. doi: 10.3389/fpls.2023.1039211 (PMC10040661; doi:10.3389/fpls.2023.1039211)

**Figure S1**. Metaphase 1 cells showing 8 bivalents (2n=16) in the Azerbaijani pomegranate cultivars: a-# Gizili, b-# Puroursid, c-#Goynar, d-#Valas, e-#Achygdona, f-#Fatima. Scale bar 10μm.

a.
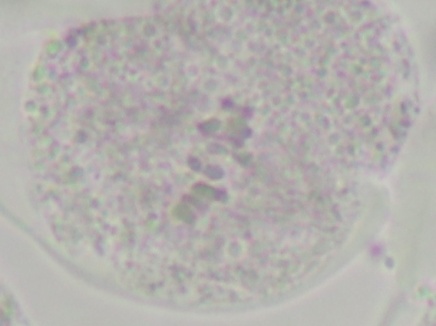
 b.
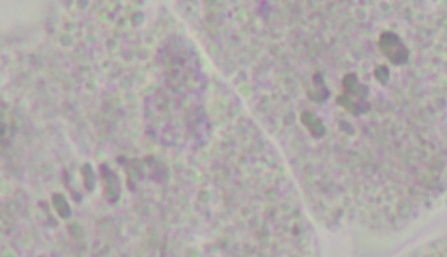
 c.
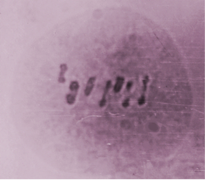


d.
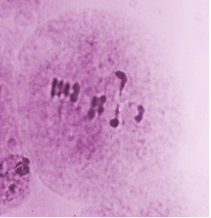
 e.
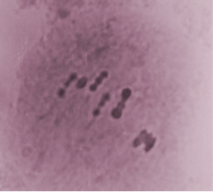
 f.
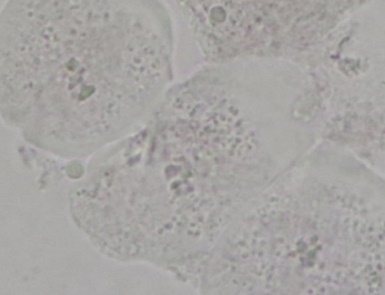

Supplement: Supplementary file 1 [file Table_1.docx]
