# Supplementary material for: Two major chromosome evolution events with unrivaled conserved gene content in pomegranate
Source: Front Plant Sci. 2023 Mar 13;14:1039211. doi: 10.3389/fpls.2023.1039211 (PMC10040661; doi:10.3389/fpls.2023.1039211)
Supplement: Supplementary file 2 [file Table_2.docx]

**Figure S2**. Boxplots showing the cross-validation (CV) values of the ADMIXTURE analysis for K ranging from 2 to 10.
