# Supplementary material for: Two major chromosome evolution events with unrivaled conserved gene content in pomegranate
Source: Front Plant Sci. 2023 Mar 13;14:1039211. doi: 10.3389/fpls.2023.1039211 (PMC10040661; doi:10.3389/fpls.2023.1039211)
Supplement: Supplementary file 3 [file Table_3.docx]

**Table S1:** Details about Pomegranate genotypes, tissue type and sequencing performed

| **RNA-Seq** |  |  |
| --- | --- | --- |
| **Genotype** | **Tissue** | **Raw paired end reads** |
| Azerbaijan guloyshasi | Leaf | 86,165,253 |
| Azerbaijan guloyshasi | New-Stem | 33,993,829 |
| Azerbaijan guloyshasi | Old-Stem | 34,663,476 |
| Azerbaijan guloyshasi | Petal | 35,691,658 |
| Azerbaijan guloyshasi | Root | 29,115,161 |
| Azerbaijan guloyshasi | Flower | 110,718,130 |
|  | **Total reads** | **330,347,507** |
| **Genome Sequencing** |  |  |
| **Genotype** | **Library preparation** | **Raw paired end reads** |
| Azerbaijan guloyshasi | Genome sequening - mate pair | 1,023,324,211 |
| Azerbaijan guloyshasi | Genome sequening - paired end 350 bp | 225,111,979 |
| Azerbaijan guloyshasi | Genome sequening - paired end 550 bp | 528,462,978 |
| Gizili - Azerbaijan | Genome sequening - paired end | 24,645,414 |
| Puroursid - USA | Genome sequening - paired end | 90,005,329 |
| Goynar - Azerbaijan | Genome sequening - paired end | 24,539,311 |
| Valas - Azerbaijan | Genome sequening - paired end | 12,455,439 |
| Achygdona - Uzbekistan | Genome sequening - paired end | 11,598,413 |
| Fatima - Azerbaijan | Genome sequening - paired end | 37,253,361 |
