# Supplementary material for: Two major chromosome evolution events with unrivaled conserved gene content in pomegranate
Source: Front Plant Sci. 2023 Mar 13;14:1039211. doi: 10.3389/fpls.2023.1039211 (PMC10040661; doi:10.3389/fpls.2023.1039211)
Supplement: Supplementary file 4 [file Table_4.docx]

**Table S2**. Overview of genome sequencing and RNA-Seq outputs and assembly. Secondary assembly for genome sequencing involves only scaffolds with length > 500 bp.

|  | **Genome** | **RNA-Seq** |  |
| --- | --- | --- | --- |
| **Primary assembly** | | | |
| Total number of filtered reads | 1,937,829,136 | 660,695,014 |  |
| Total number of reads in contig assembly | 1,779,348,905 | 468,338,238 |  |
| Total number of contigs | 908,448 | 78,359 |  |
| N50 | 1,568 | 1,657 |  |
| Total base pairs | 385,671,337 | 67,784,898 |  |
| Total base pairs without 'N' | 385,671,337 | 65,888,698 |  |
| **Secondary assembly** | | | |
| Total number of scaffolds | 12,363 | 58,064 |  |
| N50 | 220,197 | 1,561 |  |
| Total base pairs | 371,603,727 | 56,497,814 |  |
| Total base pairs without 'N' | 270,633,515 | 55,446,145 |  |
| Repeat region % of assembly | 48.2% | . |  |
