# Supplementary material for: Two major chromosome evolution events with unrivaled conserved gene content in pomegranate
Source: Front Plant Sci. 2023 Mar 13;14:1039211. doi: 10.3389/fpls.2023.1039211 (PMC10040661; doi:10.3389/fpls.2023.1039211)
Supplement: Supplementary file 5 [file Table_5.docx]

**Table S3**. Variants (SNPs and INDELs) between re-sequenced cultivars and Tunisia genome

| **Cultivar** | **Variants** | **SNPs** | **INDELs** |
| --- | --- | --- | --- |
| **Achygdona-UZB** | 208,041 | 175,242 | 32,799 |
| **Fatima-AZR** | 305,923 | 255,512 | 50,411 |
| **Gizili-AZR** | 270,830 | 223,123 | 47,707 |
| **Goynar-AZR** | 271,648 | 220,286 | 51,362 |
| **Puroursid-USA** | 323,685 | 255,093 | 68,592 |
| **Valas-AZR** | 232,743 | 195,544 | 37,199 |
| **Total** | 648,470 | 579,648 | 68,822 |
